# Supplementary material for: Super-resolution label-free volumetric vibrational imaging
Source: Nat Commun. 2021 Jun 15;12:3648. doi: 10.1038/s41467-021-23951-x (PMC8206358; doi:10.1038/s41467-021-23951-x)
Supplement: Supplementary file 1 — Supplementary Information [file 41467_2021_23951_MOESM1_ESM.pdf]

Supplementary Information for

Super-Resolution Label-free Volumetric Vibrational Imaging

Chenxi Qian†[1], Kun Miao†[1], Li-En Lin[1], Xinhong Chen[2], Jiajun Du[1] and Lu Wei*[1]

1. Division of Chemistry and Chemical Engineering, California Institute of Technology, Pasadena, California 91125, United States
2. Division of Biology and Biological Engineering, California Institute of Technology, Pasadena, California 91125, USA

† These authors contributed equally: Chenxi Qian, Kun Miao

*E-mail: lwei@caltech.edu

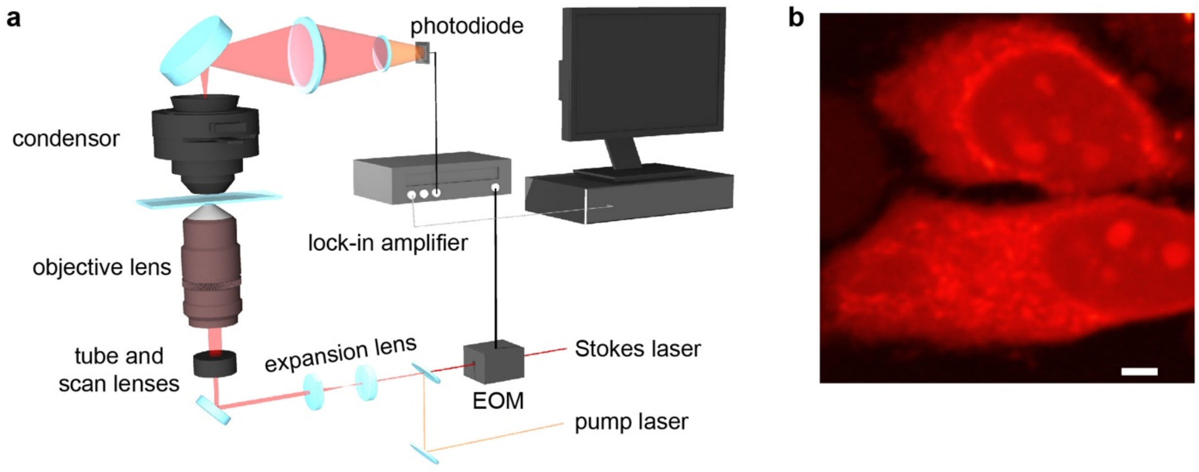

Supplementary Figure 1. Setup for the stimulated Raman scattering (SRS) microscopy. (a) Instrumental setup of SRS microscope. EOM: Electro-Optic Modulator. When the energy difference between the Pump laser photons and the Stokes laser photons matches with the vibrational frequency of target chemical bonds (e.g. 2940 cm^{-1} for the symmetric vibrational motion of CH_3), the chemical bonds are efficiently driven from the vibrational ground state to the vibrational excited state, creating stimulated Raman loss in pump beam, which is subsequently detected by a photodiode and provides the imaging contrast. (b) A representative regular-resolution SRS image of HeLa cells targeting the protein CH_3 vibration at 2940 cm^{-1}. Scale bar: 3 μm.

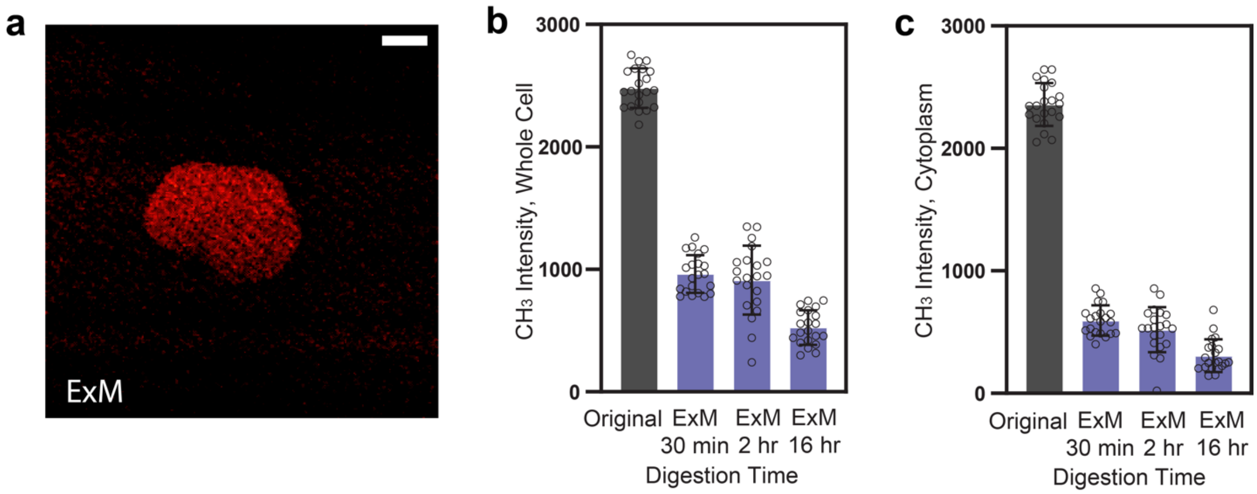

Supplementary Figure 2. SRS quantification of protein loss following the ExM protocol. (a) Contrast-enhanced SRS image for Fig. 1c with a 2-time enhancement on the intensity scale. Scale bar: 20 µm. The length scale is in terms of distance after sample expansion. (b)-(c) Quantification of protein retention level on different protease digestion time by proteinase K on whole cells (b) and from the cytoplasm (c). SRS images of protein CH_3 from HeLa cells were acquired after designated digestion times. The protein signals decrease rapidly upon protease treatment, confirming that the digestion step in ExM causes significant protein loss. For each digestion time, n=21 cells were examined over 3 independent experiments. Data shown as mean ± SD.

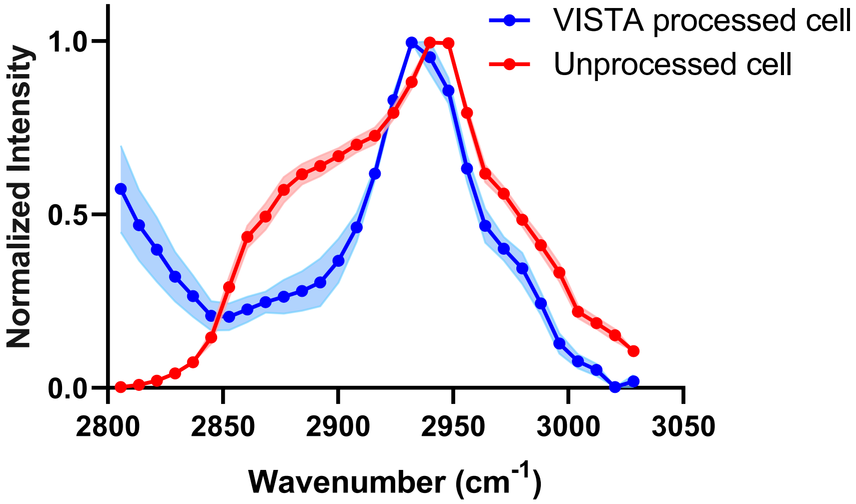

Supplementary Figure 3. Hyperspectral SRS from HeLa cells with and without VISTA processing. The spectra were acquired by sweeping pump laser wavelength with 0.5 nm step size from 783.3 to 799.8 nm, with Stokes laser fixed at 1031.2 nm. The evident loss of lipid peak at 2855 cm^{-1} between normalized SRS spectra from unprocessed cells (red) and VISTA processed cells (blue) confirms the loss of lipid content from the protein denaturation step, which washes out the lipids. The intact peak shape for CH_3 at 2940 cm^{-1} confirms that VISTA retains proteins. The shoulder around 2800 cm^{-1} for VISTA processed cell spectrum (blue) originates from D_2O. VISTA processed cell, N=10; unprocessed cell, N=15. Data shown as mean ± SD.

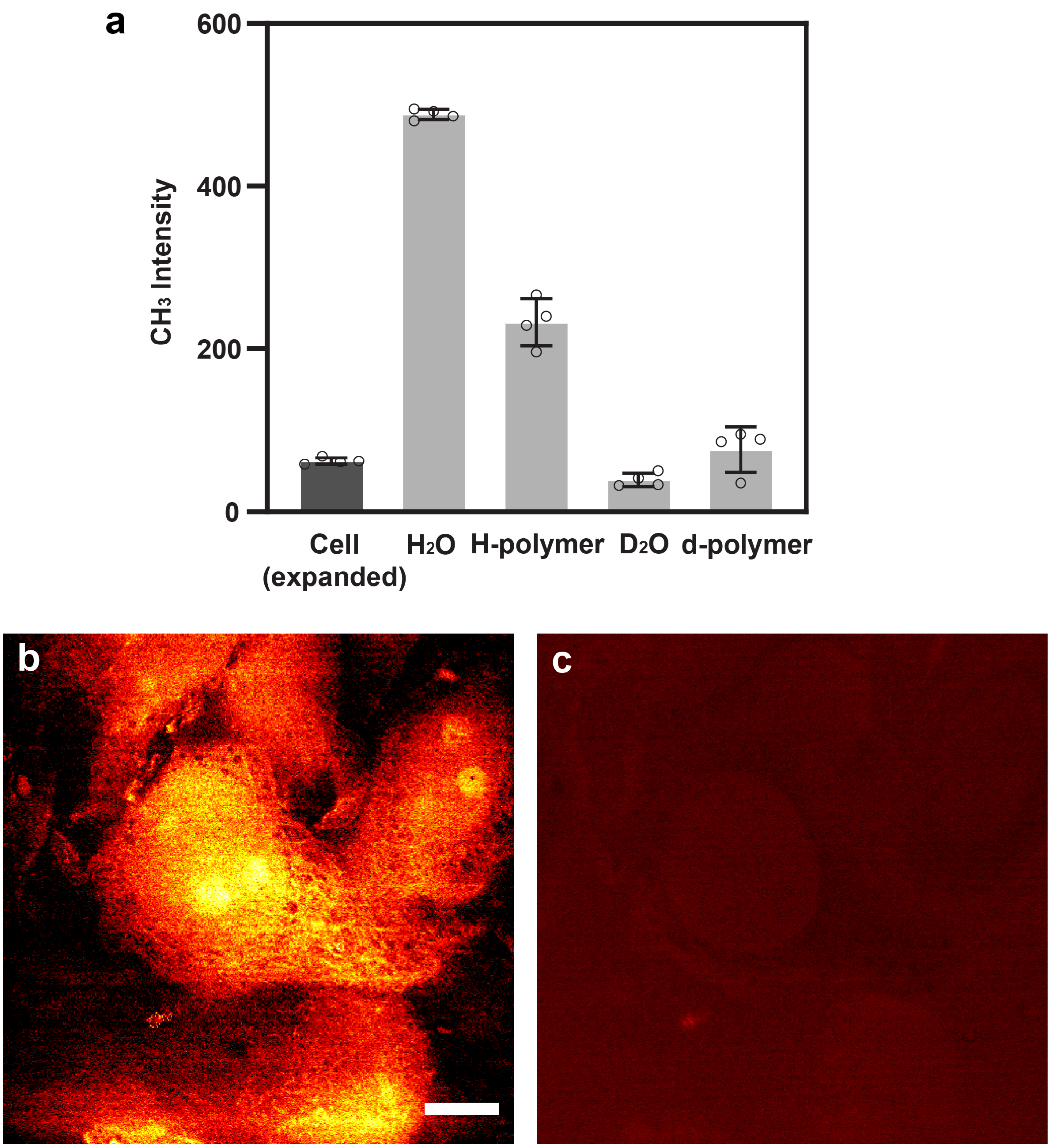

Supplementary Figure 4. Quantification of backgrounds in VISTA CH_3 images on HeLa cells. (a) Background contributions from each component (i.e. O-H stretch tail from H_2O and C-H vibrations from the polymers composed of sodium acrylate and acrylamide monomers) were obtained by comparing normal VISTA cell images with VISTA images using corresponding deuterated components. The change of the backgrounds is constant across field of views after deuteration. For each component, n=4 samples were examined over 4 independent experiments. Data shown as mean ± SD. (b-c) Representative VISTA images from expanded HeLa cells embedded in the deuterated polymer at the CH_3, 2940 cm^{-1} channel for cellular distributions (b) and the corresponding CD, 2176 cm^{-1} channel for deuterated polymers (c). Scale bar: 20 μm

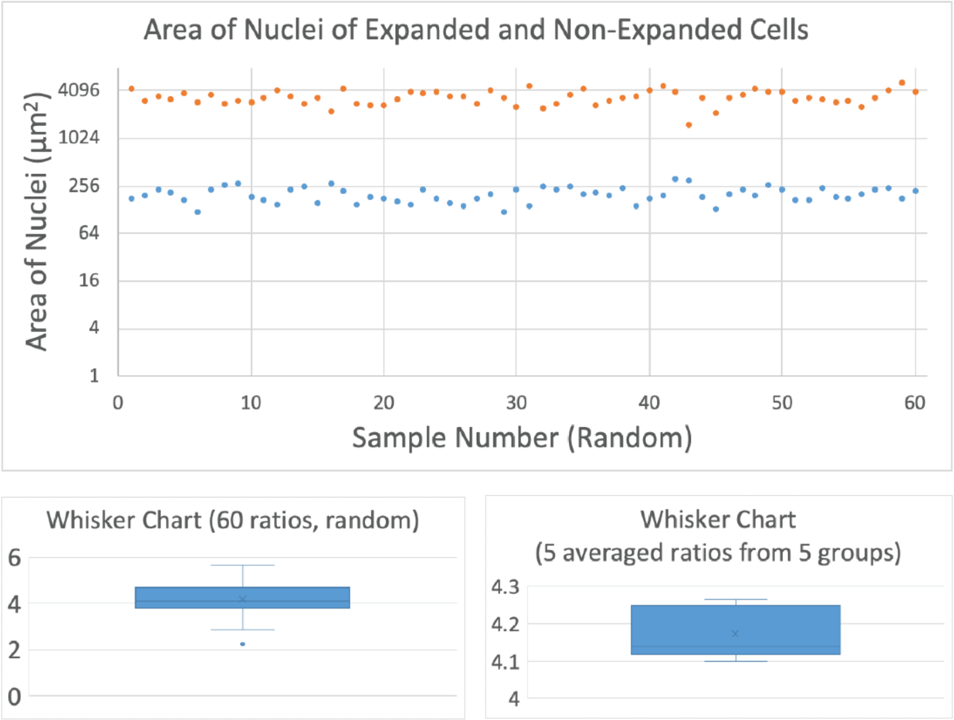

Supplementary Figure 5. Statistical quantification of the average expansion ratio for cell samples by VISTA.[1] Top: Areas of randomly chosen 60 expanded (orange) and non-expanded (blue) cells across 5 individual sample groups. Bottom: statistics with whisker charts for calculation of the average expansion ratios on cells. The left whisker chart (4.2±0.7) is calculated based on total 60 random expanded vs non-expanded pairs, and the right one (4.2±0.1) is calculated based on 5 averaged ratios from the 5 sample groups (12 cells in each group). For the box plots, top/bottom of vertical line is the maxima/minima, center line is median, x in the box is mean, and the top/bottom of box is median of the top/bottom half (3rd quartile/1st quartile).

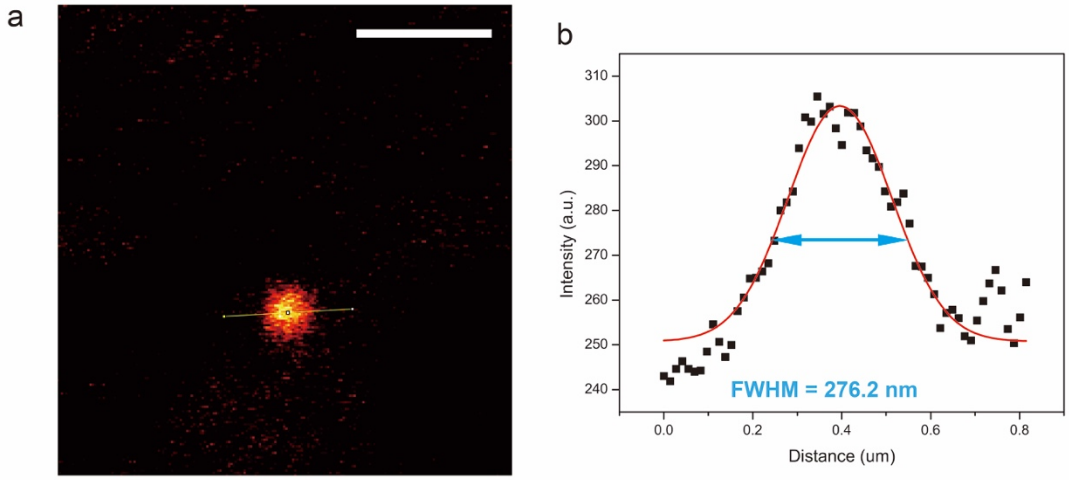

Supplementary Figure 6. Quantification of SRS resolution under a higher numerical aperture (NA) objective lens. A representative image (a) and the corresponding fitted cross-section profile (FWHM of 276.2nm) (b) from a 100-nm polystyrene bead, by targeting the C-H vibration at 3050 cm^{-1} with a 60X water objective (Olympus, UPLSAPO60XWIR, 1.2 NA). Scale bar: 2 μm.

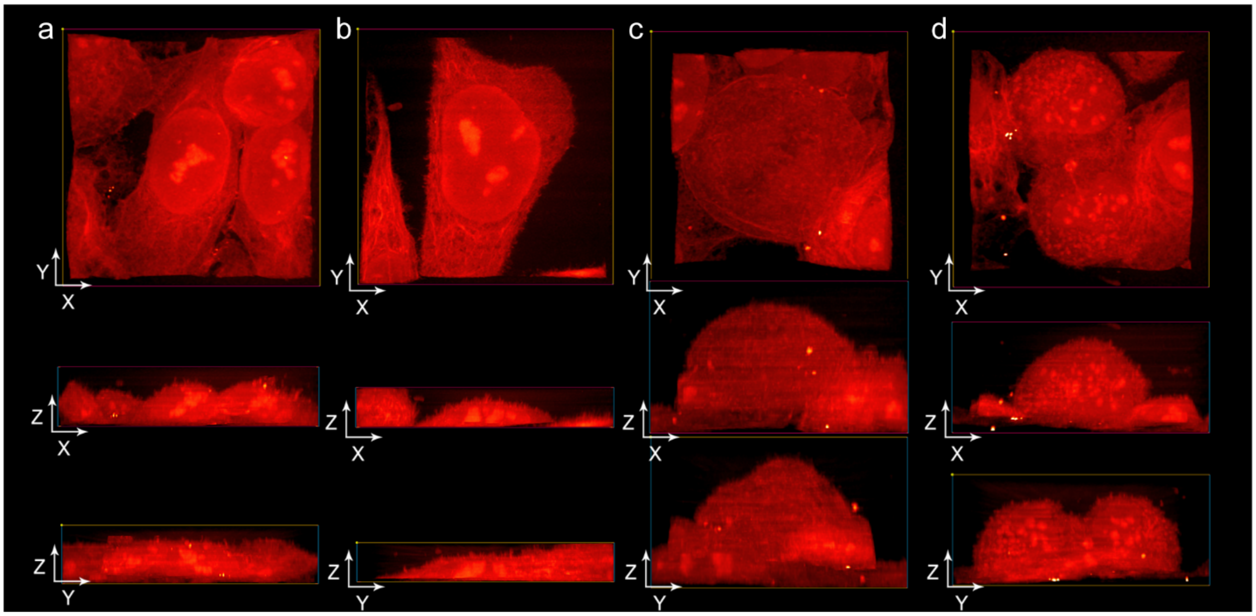

Supplementary Figure 7. High-resolution three-dimensional VISTA views of cellular morphology and subcellular structures of interphase and mitotic HeLa cells. (a-b) Volume HeLa cells at interphase. The network structure in the cytoplasm is clear. In the x-z view, minor upward extrusions from cell surface (zx view) were also captured. (c-d) Volume HeLa cells during mitosis for single-z images shown in Fig. 2b and Fig. 2d.

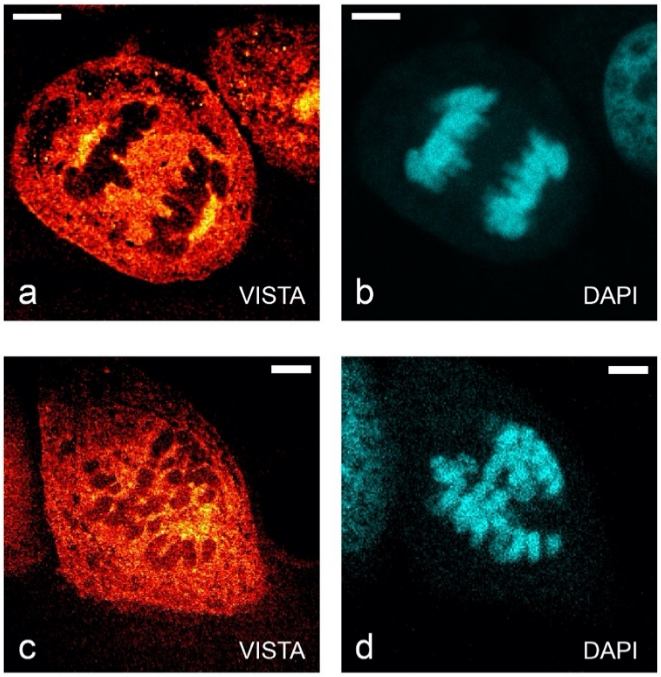

Supplementary Figure 8. Correlative VISTA images and the fluorescent images with DAPI stain on mitotic HeLa cells. Fluorescent DAPI stains confirms that the dark regions shown in VISTA images on anaphase (a-b) and prophase (c-d) HeLa cells are chromosomes with a low CH_3 signals (i.e. low protein contents). Scale bars: 20 µm. The length scale is in terms of distance after sample expansion.

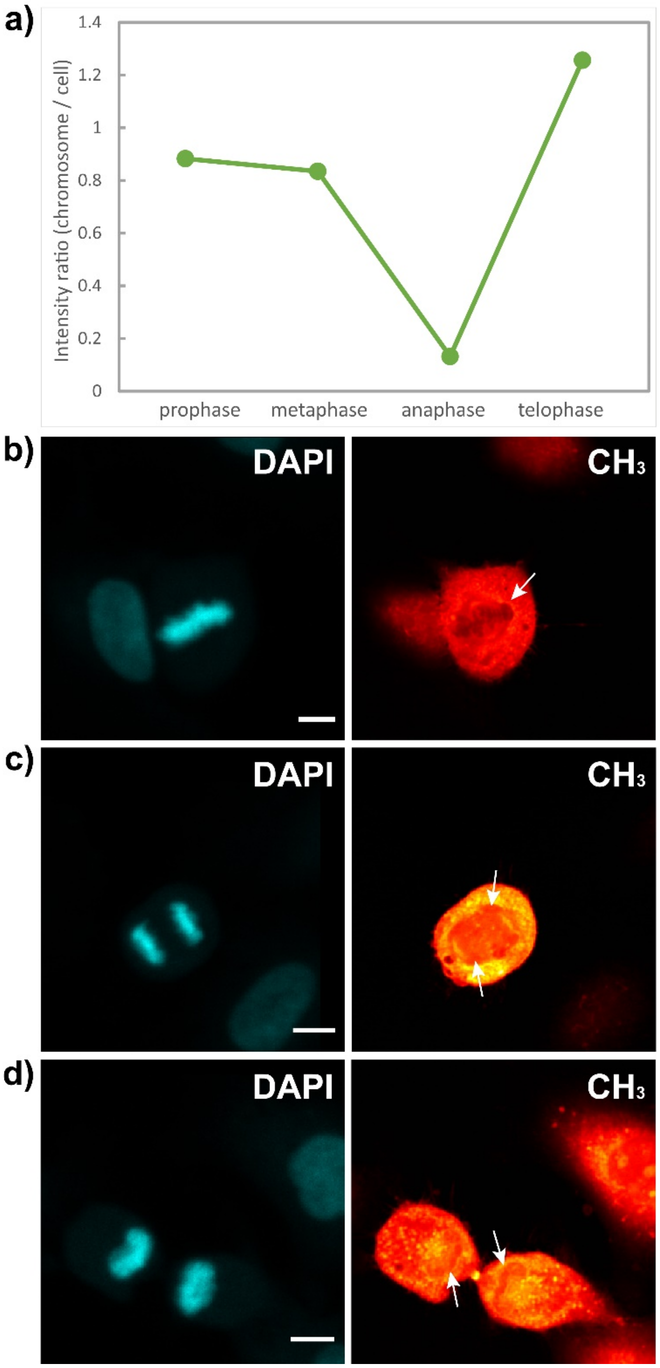

Supplementary Figure 9. Confirmation of protein abundance change in the chromosomal regions in HeLa cells throughout mitosis. (a) Quantification of relative protein abundance in chromosomal regions in reference to the protein CH_3 signals from the whole cells (i.e. the chromosome/cell ratio) for expanded HeLa cells across different stages of mitosis, shown in Fig. 2a-d. (prophase: 0.88; metaphase: 0.83; anaphase: 0.14; telophase: 1.26). (b-d) SRS images (CH_3, 2940 cm^{-1}) and correlative DAPI-stain fluorescence images of PFA-fixed but unprocessed mitotic HeLa cells in metaphase (b); anaphase (c); and telophase (d). Arrows indicate corresponding chromosome regions in the SRS images guided by DAPI fluorescence stain. Scale bars: 10 µm.

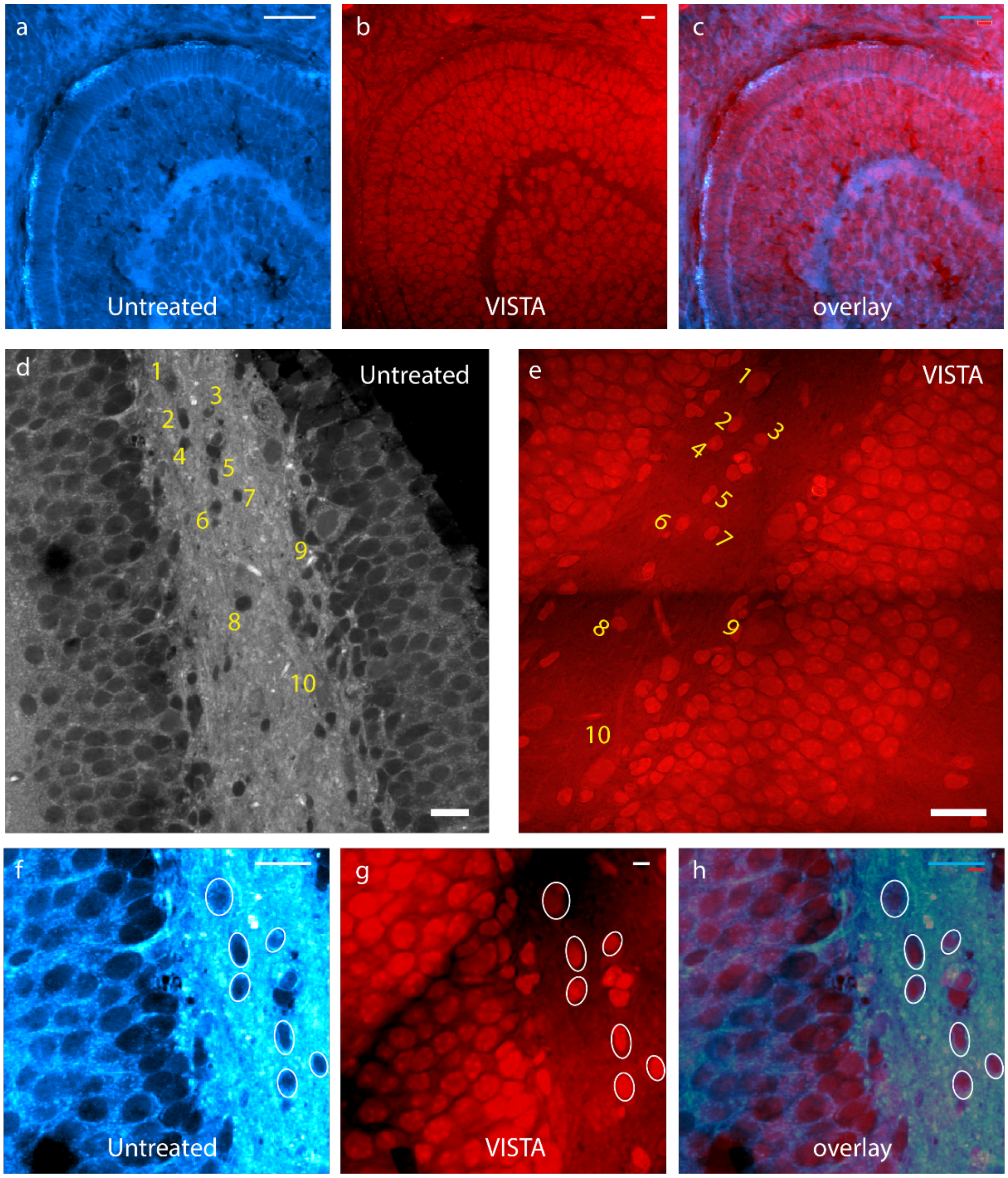

Supplementary Figure 10. Imaging registrations for tissues before and after expansion. (a-c) SRS imaging of an untreated zebrafish retina tissue (a), VISTA imaging of the same sample (b), and the overlay (e). (d-e) SRS imaging of an untreated mouse brain tissue (a) and VISTA image of the same sample (b), with labels of 9 correlating features for expansion ratio calculation. (f-h) SRS imaging of an untreated brain tissue (a), VISTA imaging of the same sample (b), and the overlay (e). White circles indicate cell bodies to guide the registration. Scale bars: 20 μm (a-d,f-h),80 μm (e).

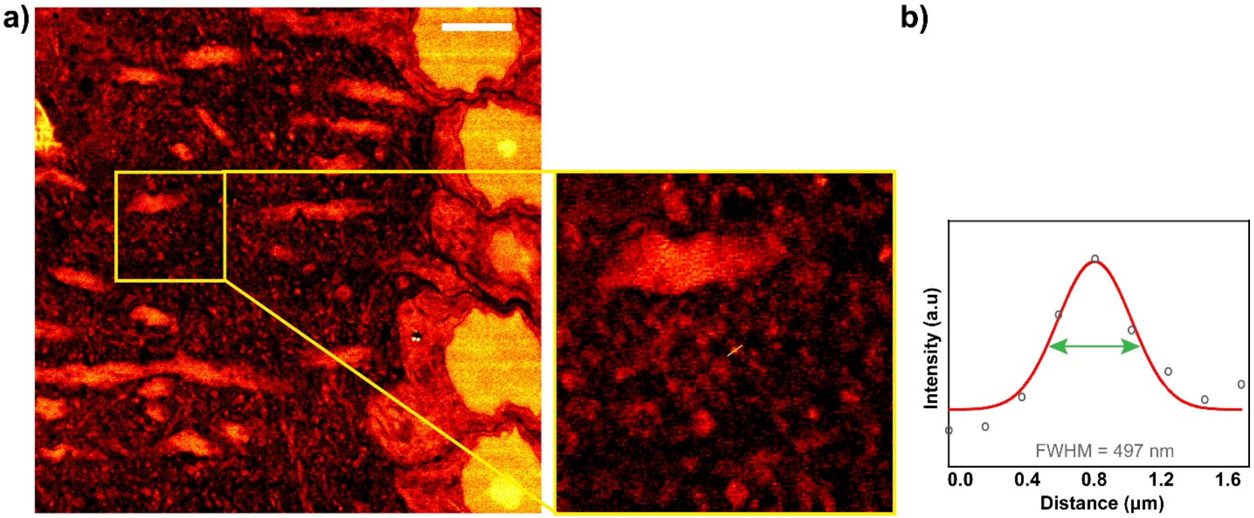

Supplementary Figure 11. Characterizations of fine dendritic structures by VISTA. (a) A representative VISTA image at the CA1 region of a mouse hippocampus sample with a zoomed-in view of fine structural details in the yellow box. (b) The cross-section profile of a dendritic structure with a FWHM of 497 nm (corresponding to a lateral size of ~175 nm for the unexpanded sample). Scale bars: 20 μm.

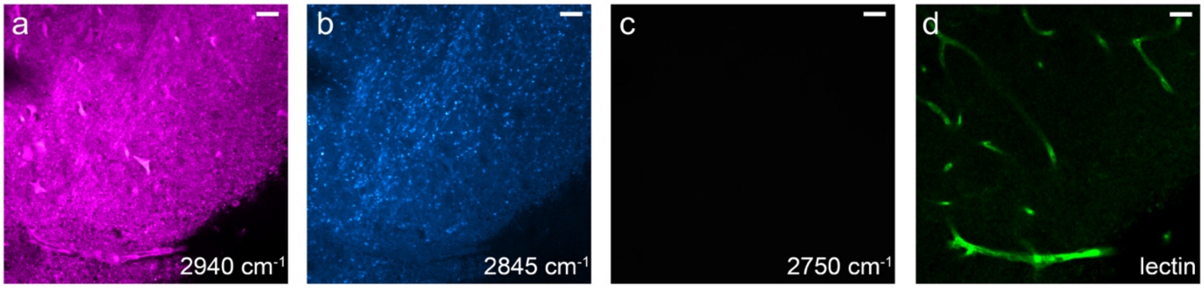

Supplementary Figure 12. Regular-resolution SRS imaging of a PFA-fixed but unprocessed mouse brain tissue. a-c, SRS imaging at 2940 cm^{-1} (a, CH_3), 2845 cm^{-1} (b, CH_2), and 2750 cm^{-1} (c, off-resonance image). Cells, processes and vessels could not be clearly identified from these SRS images. d, fluorescence image of lectin-stained blood vessel on the same tissue. Scale bars: 20 μm.

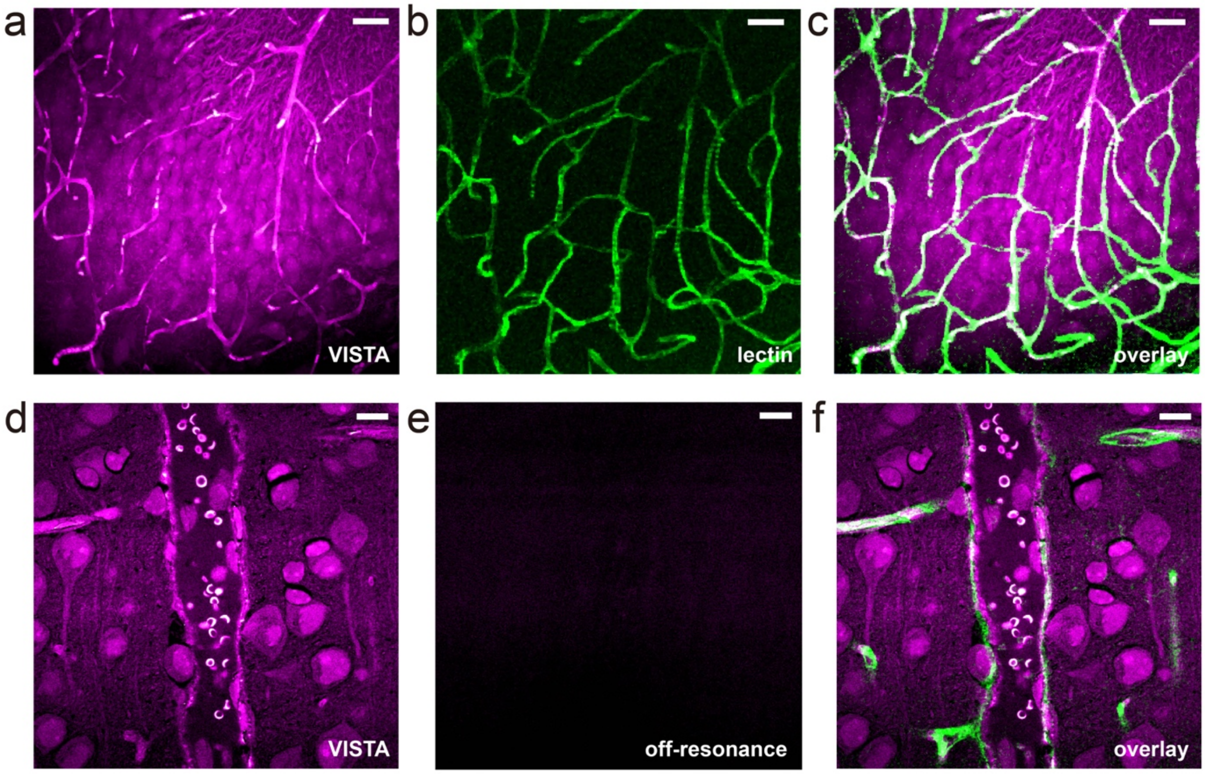

Supplementary Figure 13. Correlative VISTA images and the fluorescent images with lectin-stained vessels in mouse brain tissues. (a-c) The overlay image (c) for parallel images of VISTA (a, shown in Fig. 3a) and fluorescence from lectin-DyLight594 stained blood-vessels (b, shown in Fig. 3b). (d-e) VISTA image of a larger vessel (likely an artery) showing red blood cells (d, shown in Fig. 3c) and the corresponding off-resonance image at 2810 cm^{-1} confirms that signals in the VISTA image (including that of the red blood cells) are exclusively SRS signals. (f) The overlay image for VISTA (Fig. 3c) and lectin-DyLight594 stained fluorescence (Fig. 3d). Scale bars: 40 μm. The length scale is in terms of distance after sample expansion.

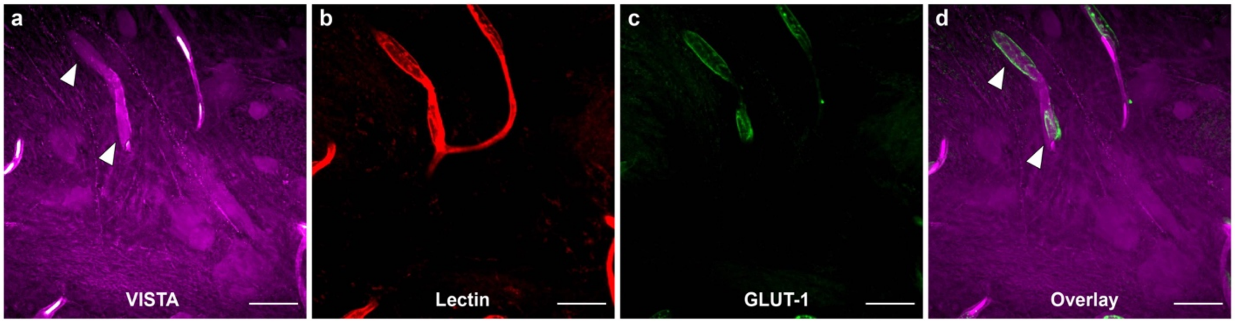

Supplementary Figure 14. Immuno-fluorescence reveals the cells in VISTA other than neuronal cells to be vascular endothelial cells. Elongated elliptical nuclei shown in VISTA (a, white arrow-headed, VISTA) co-localize (d, Overlay) with both fluorescent lectin-stained vessels (b, Lectin), and GLUT-1 (c, glucose transporter 1, brain endothelial cell marker) immuno-fluorescence-stained brain vascular endothelial cells. Images are shown as maximum Z projection. Scale bars: 50 μm. The length scale is in terms of distance after sample expansion.

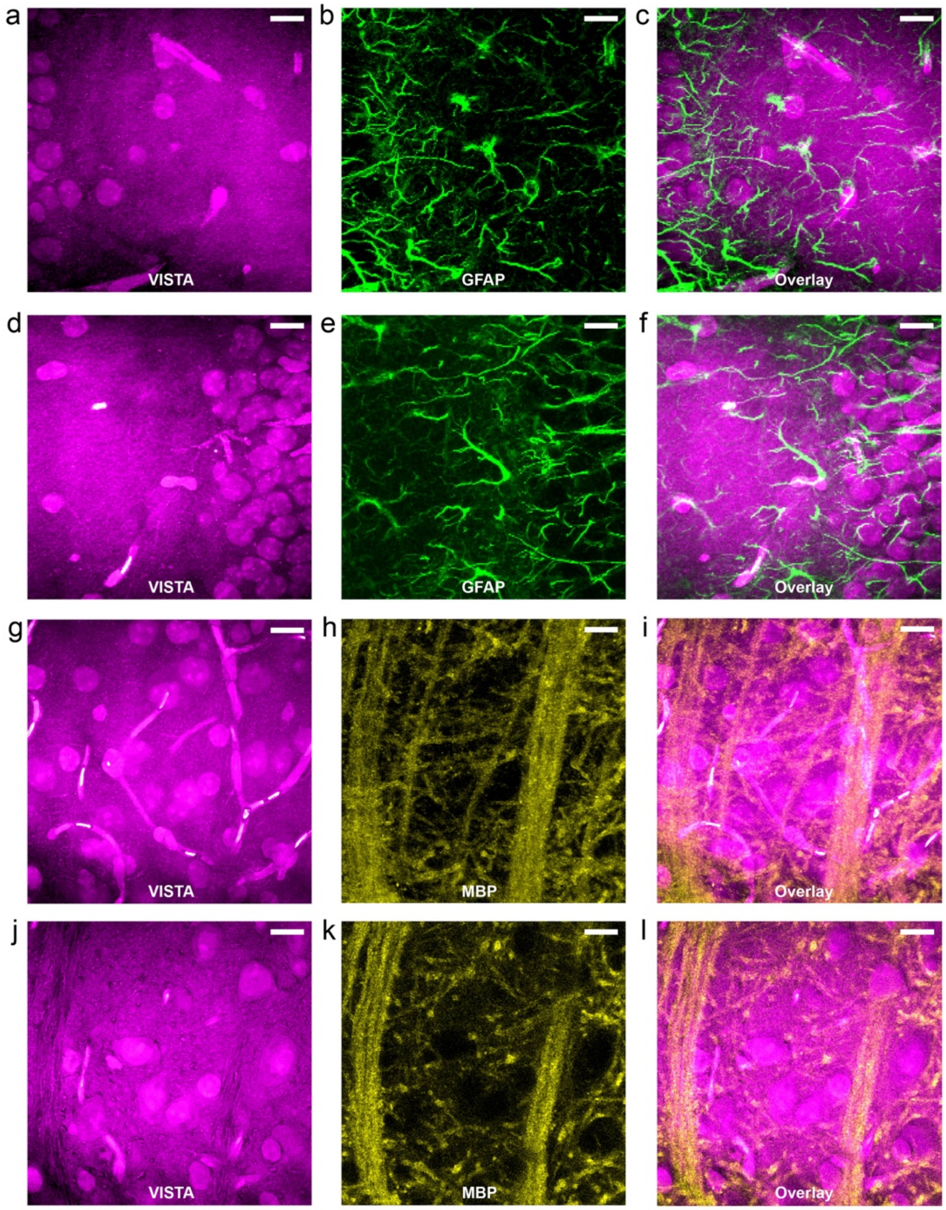

Supplementary Figure 15. Immunofluorescence confirms the absence of cytoplasmic structures from astrocytes and oligodendrocytes in VISTA. (a-f) Maximum z projection of VISTA image (a, d), immuno-stained fluorescence image with GFAP (Glial Fibrillary Acidic Protein, the astrocyte cellular maker) antibodies (b, e) and the overlay (c, f). (g-i) maximum z projection of VISTA image (g), immuno-stained fluorescence image with MBP (Myelin Basic Protein, the myelin and oligodendrocyte cellular maker) antibodies (h) and the overlay (i). (j-l) single-slice VISTA image (j), immuno-stained fluorescence image with MBP antibodies (k) and the overlay (l). No correlation is identifiable between VISTA revealed cytoplasmic structures and GFAP or MBP stained cellular fluorescence images. Scale bars: 40 μm. The length scale is in terms of distance after sample expansion.

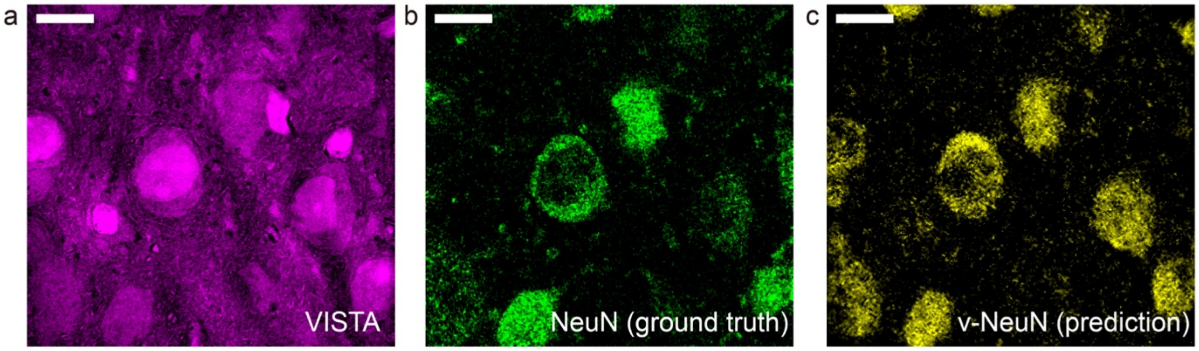

Supplementary Figure 16. Label-free VISTA prediction for neuronal cell bodies in brain tissues. a-c, The input VISTA image (a), the ground truth fluorescence image of NeuN stained matured neurons (b) and the predicted VISTA-NeuN (v-NeuN) image of matured neurons from a (c). Scale bars: 40 μm. The length scale is in terms of distance after sample expansion.

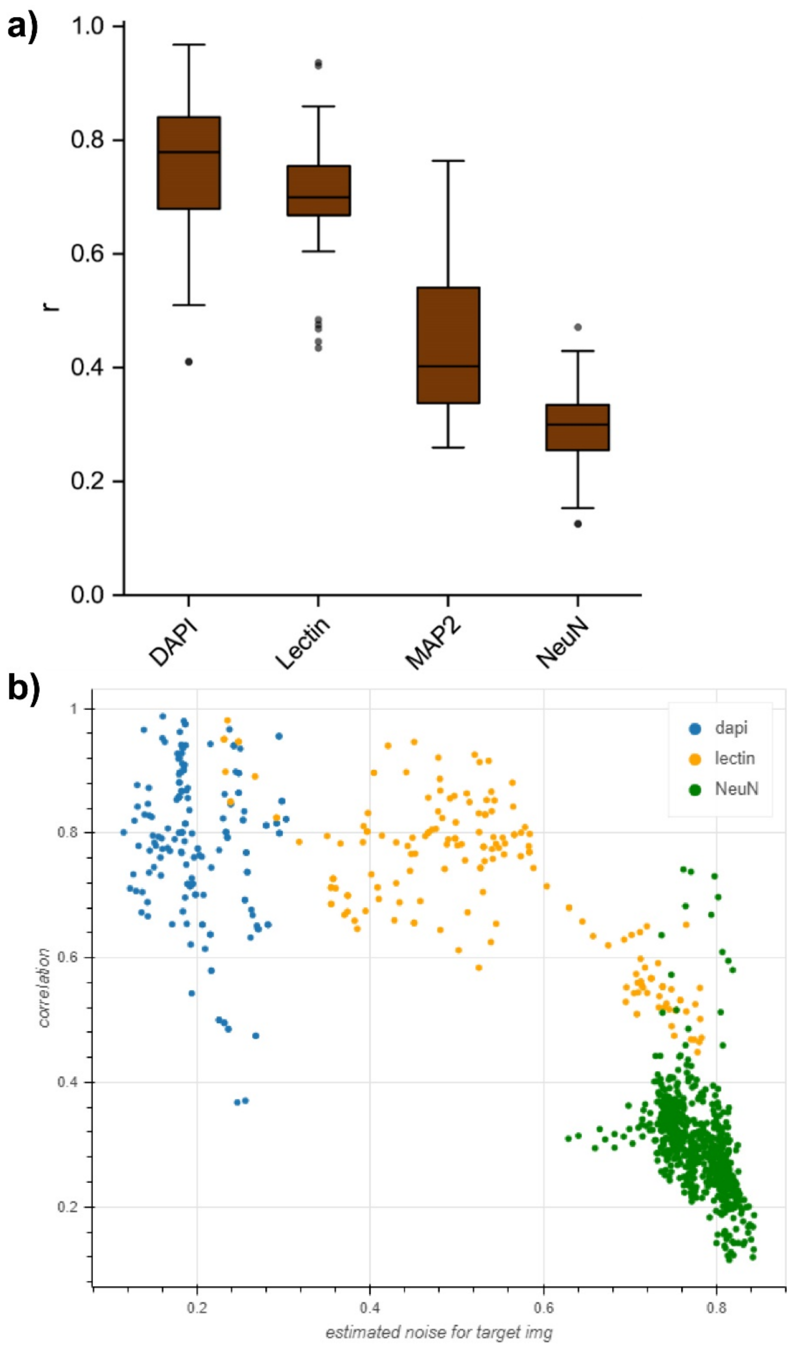

Supplementary Figure 17. Model performance for the U-Net models, quantified by Pearson correlation coefficient (r). (a) Each point in the plot represents a target/predicted image pair. The boxes indicate the 25th, 50th, and 75th percentile of the Pearson's r for each model, with whiskers with maximum 1.5 interquartile range. Points within the box are not shown. DAPI, N=48; Lectin, N=37; MAP2, N=54; NeuN, N=130. (b) Correlation between the Pearson's r for the prediction results and the estimated standard deviation of the noise for ground-truth fluorescence images for DAPI, Lectin and NeuN.

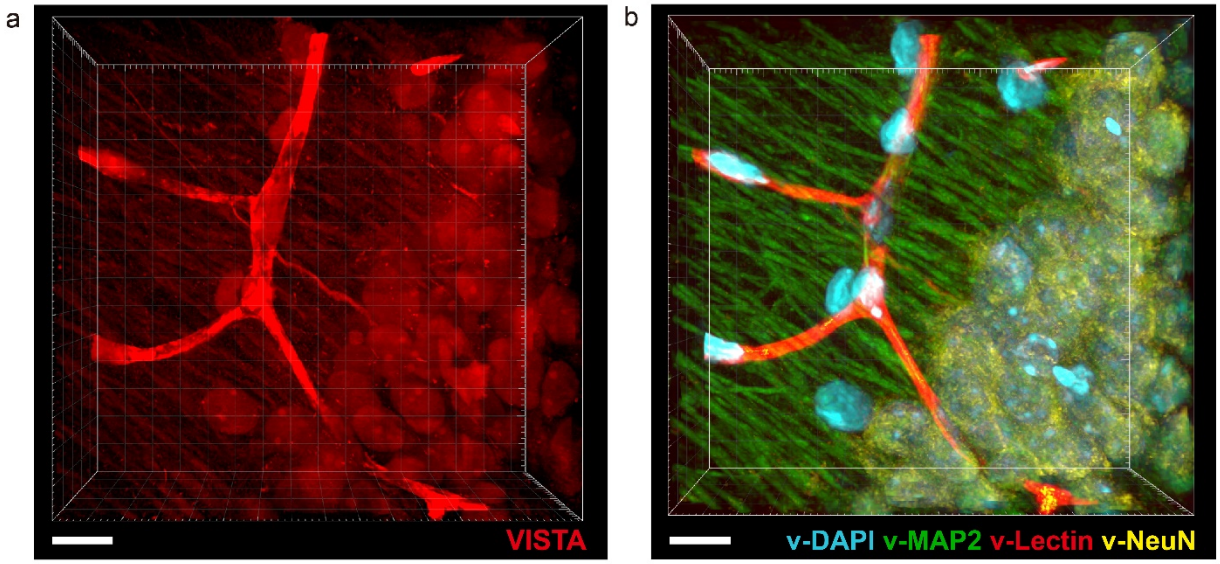

Supplementary Figure 18. Label-free VISTA prediction for multi-component imaging of mouse hippocampus. (a) The input VISTA image; (b) the predicted multicolor image with predicted v-DAPI (cyan), v-MAP2 (green), v-lectin (red) and v-NeuN (yellow) components, as shown in Fig. 4j. Scale bars: 40 µm. The length scale is in terms of distance after sample expansion.

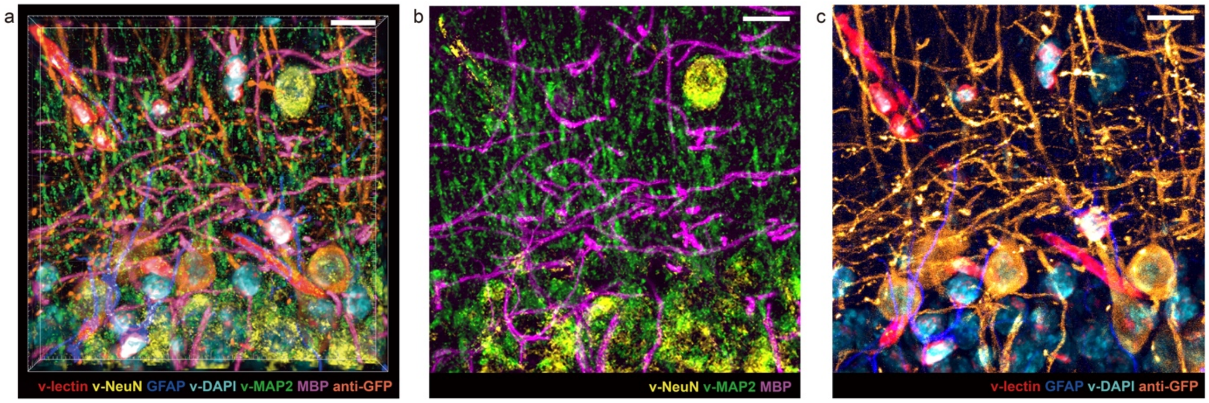

Supplementary Figure 19. 7-color multiplex imaging combining both label-free VISTA prediction and fluorescence imaging in hippocampus of Thy1-YFP mouse. (a) Volume 3D presentation of 7-color overlay image. (b-c) Maximum z projection view of 7 components in two sets of 3-color (b) and 4-color (c) overlay. VISTA components: v-NeuN (yellow), v-MAP2 (green), v-DAPI (cyan), v-lectin (red); immuno-fluorescence components: GFAP (blue), MBP (magenta) and anti-GFP (orange). Scale bars: 40 µm. The length scale is in terms of distance after sample expansion.

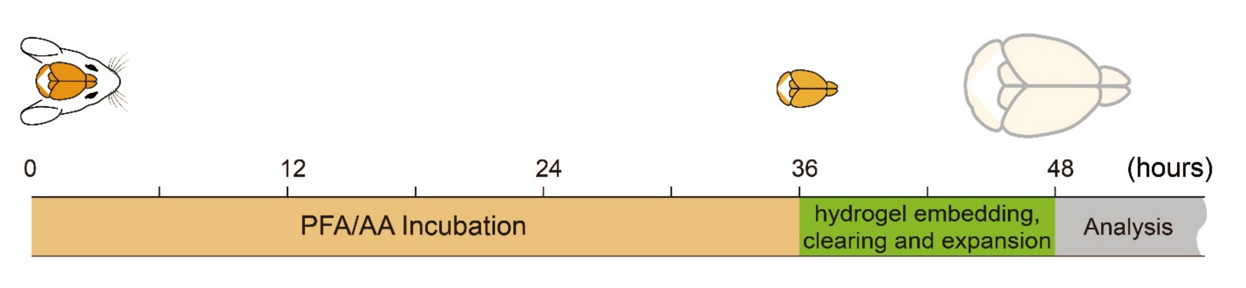

Supplementary Figure 20. Workflow of the sample processing steps for VISTA. Typical sample processing steps were completed with a 48-hour for mouse brain tissues before VISTA analysis. (PFA: paraformaldehyde; AA: acrylamide)

Reference:

1. Büttner, M. *et al.* Challenges of Using Expansion Microscopy for Super-resolved Imaging of Cellular Organelles. *ChemBioChem* **22**, 686–693 (2021).
