# Supplementary material for: Super-resolution label-free volumetric vibrational imaging
Source: Nat Commun. 2021 Jun 15;12:3648. doi: 10.1038/s41467-021-23951-x (PMC8206358; doi:10.1038/s41467-021-23951-x)
Supplement: Supplementary file 3 — Reporting Summary [file 41467_2021_23951_MOESM3_ESM.pdf]

nature research

Corresponding author(s): Lu Wei

Last updated by author(s): Apr 26, 2021

Reporting Summary

Nature Research wishes to improve the reproducibility of the work that we publish. This form provides structure for consistency and transparency in reporting. For further information on Nature Research policies, see our Editorial Policies and the Editorial Policy Checklist.

Statistics

For all statistical analyses, confirm that the following items are present in the figure legend, table legend, main text, or Methods section.

n/a	Confirmed	
[ ]	[x]	The exact sample size (n) for each experimental group/condition, given as a discrete number and unit of measurement
[ ]	[x]	A statement on whether measurements were taken from distinct samples or whether the same sample was measured repeatedly
[x]	[ ]	The statistical test(s) used AND whether they are one- or two-sided<br>*Only common tests should be described solely by name; describe more complex techniques in the Methods section.*
[ ]	[x]	A description of all covariates tested
[ ]	[x]	A description of any assumptions or corrections, such as tests of normality and adjustment for multiple comparisons
[ ]	[x]	A full description of the statistical parameters including central tendency (e.g. means) or other basic estimates (e.g. regression coefficient) AND variation (e.g. standard deviation) or associated estimates of uncertainty (e.g. confidence intervals)
[x]	[ ]	For null hypothesis testing, the test statistic (e.g. F, t, r) with confidence intervals, effect sizes, degrees of freedom and P value noted<br>*Give P values as exact values whenever suitable.*
[x]	[ ]	For Bayesian analysis, information on the choice of priors and Markov chain Monte Carlo settings
[x]	[ ]	For hierarchical and complex designs, identification of the appropriate level for tests and full reporting of outcomes
[ ]	[x]	Estimates of effect sizes (e.g. Cohen's d, Pearson's r), indicating how they were calculated

Our web collection on statistics for biologists contains articles on many of the points above.

Software and code

Policy information about availability of computer code

Data collection	16-bit grey scale images for VISTA and immunofluorescence images are acquired by Olympus Fluoview 3000 software.
Data analysis	We used MATLAB (R2018b), ImageJ (1.52p), Python (3), OriginPro (8.5), and Imaris Viewer (9.7) to perform the analyses. The U-Net training was performed using the Adam optimizer (Version 1) to optimize the mean squared error between the fluorescence image and the predicted image. MATLAB code used for PSF determination and Python code for U-Net training and prediction in this paper is available at https://github.com/Li-En-Good/VISTA.

For manuscripts utilizing custom algorithms or software that are central to the research but not yet described in published literature, software must be made available to editors and reviewers. We strongly encourage code deposition in a community repository (e.g. GitHub). See the Nature Research guidelines for submitting code & software for further information.

Data

Policy information about availability of data

All manuscripts must include a data availability statement. This statement should provide the following information, where applicable:

- Accession codes, unique identifiers, or web links for publicly available datasets
- A list of figures that have associated raw data
- A description of any restrictions on data availability

The authors declare that all data supporting the findings of the present study are available in the article and its supplementary figures and tables, or from the corresponding author upon request.

Field-specific reporting

Please select the one below that is the best fit for your research. If you are not sure, read the appropriate sections before making your selection.

- [x] Life sciences
- [ ] Behavioural & social sciences
- [ ] Ecological, evolutionary & environmental sciences

For a reference copy of the document with all sections, see nature.com/documents/nr-reporting-summary-flat.pdf

Life sciences study design

All studies must disclose on these points even when the disclosure is negative.

Sample size	No sample size calculation was performed. Number of experimental was chosen based on previous experiments using similar experimental settings. For imaging experiments, a sample size of at least 3 was used as the minimum samples for quantification. The specific sample size is indicated in the manuscript.
Data exclusions	No data excluded
Replication	Experiments were performed with three different biological replicates and at least two technical replicates. Attempts at replication are successful. VISTA on HeLa cells, zebrafish embryos, and mouse brain were achieved more than 5 times on independent samples.
Randomization	Random allocation.
Blinding	Blinding was not relevant to this study because this study did not involve clinical sample grouping etc.

Reporting for specific materials, systems and methods

We require information from authors about some types of materials, experimental systems and methods used in many studies. Here, indicate whether each material, system or method listed is relevant to your study. If you are not sure if a list item applies to your research, read the appropriate section before selecting a response.

Materials & experimental systems

n/a	Involved in the study	
☐	☒	Antibodies
☐	☒	Eukaryotic cell lines
☒	☐	Palaeontology and archaeology
☐	☒	Animals and other organisms
☒	☐	Human research participants
☒	☐	Clinical data
☒	☐	Dual use research of concern

Methods

n/a	Involved in the study	
☒	☐	ChIP-seq
☒	☐	Flow cytometry
☒	☐	MRI-based neuroimaging

Antibodies

Antibodies used	Primary antibodies: anti-myelin basic protein in rat (Abcam, ab7349); anti-myelin basic protein in rabbit (Abcam, ab40390); anti-GFAP in chicken (Abcam, ab4674); anti-chicken IgY, Alexa 488 (Invitrogen, A-32931); anti-GFP Alexa Fluor 647 (Invitrogen, A-31852); anti-MAP2 (Cell Signaling Technology,8707); anti-NeuN (Cell Signaling Technology, 24307); anti-GLUT-1 (Millipore Sigma, 07-1401); Lycopersicon Esculentum Lectin DyLight®594 (Vector Laboratories, DL-1177-1). Secondary antibodies: goat anti-rat IgG, Alexa Fluor 568 (Invitrogen, A-11077); goat anti-mouse IgG, Alexa Fluor 647 (Invitrogen, A-21236); goat anti-rabbit IgG, Alexa Fluor 488 (Invitrogen, A-11034); goat anti-chicken IgY, Alexa Fluor 647 (Invitrogen, A-21449).
Validation	All antibodies used are from commercial sources as described. Only antibodies that have been validated by the vendor with in vitro and in situ experiments and/or heavily used by the community with publication in several references were used. The validation and references for each are publicly available on the respective vendor websites that can reached via the catalog numbers listed above.

Eukaryotic cell lines

Policy information about cell lines

Cell line source(s)	HeLa-CCL2 was purchased from ATCC.
Authentication	HeLa-CCL2 used was not authenticated.
Mycoplasma contamination	HeLa-CCL2 used was tested negative for mycoplasma contamination.

April 2020

Commonly misidentified lines (See ICLAC register)	No commonly misidentified cell line was used in the study.

Animals and other organisms

Policy information about studies involving animals; ARRIVE guidelines recommended for reporting animal research

Laboratory animals	The C57BL/6J (000664), Thy1-YFP mice(003709) mouse lines used in this study were purchased from the Jackson Laboratory (JAX) and bred in our animal facility. 6- to 8-week-old, male and female C57BL/6J, Thy1-YFP mice were used for tissue collection. Animals were group housed (3–4 per group) throughout the duration of the experiment in a vivarium on a 12 hr light/dark cycle (lights off at 0600 hr, lights on at 1800 hr) with ad libitum access to food and water, in rooms at 64-79 F with 30-70% humidity under pathogen-free conditions.
Wild animals	This study did not involve wild animal.
Field-collected samples	No sample were field collected.
Ethics oversight	All animal procedures performed in this study were approved by the California Institute of Technology Institutional Animal Care and Use Committee (IACUC), and we have complied with all relevant ethical regulations.

Note that full information on the approval of the study protocol must also be provided in the manuscript.
